# Supplementary material for: A genetic screen for aldicarb resistance of Caenorhabditiselegans dauer larvae uncovers 2 alleles of dach-1, a cytochrome P450 gene
Source: G3 (Bethesda). 2022 Oct 4;12(12):jkac266. doi: 10.1093/g3journal/jkac266 (PMC9713407; doi:10.1093/g3journal/jkac266)
Supplement: jkac266_Supplemental_Figure_Legends [file jkac266_supplemental_figure_legends.docx]

**Supplemental Figure legends**

Figure S1. Investigation of dauer-specific aldicarb resistant gene (A) N2 dauer (n = 27) is hypersensitive to aldicarb compared to N2 young adult (n = 28). (B) *daf-2* mutant dauer (n = 30) is hypersensitive to aldicarb compared to *daf-2* L3 animals (n = 30). (C) *daf-2;ys51* (n = 59) and *daf-2;ys52* (n = 60) L3 larvae showed similar aldicarb-sensitivity (to 1 mM aldicarb plate) with *daf-2* L3 (n = 60) larvae. Error bar indicates SE.

Figure S2. Whole genome sequencing of *dach-1* mutant. (A) Experimental scheme of F2 pooling sequencing strategy. (B) Every independent F2 homozygous was resistant to aldicarb. n > 3 for each experiment. *** P<0.001 (One-way ANOVA, Dunnett’s post-test).

Figure S3. Complementation test of *ys51* and *ys52*. They failed to complement each other, suggesting that *ys51* and *ys52* are allelic. n = 3 for each experiment. Error bar indicates SE. *** P<0.001 (One-way ANOVA, Dunnett’s post-test). Aldicarb sensitivity was measured as the ratio of paralysis at 80 min.

Figure S4. *dach-1* plays a role in the resistance of trichlorfon, an organophosphate cholinesterase inhibitor. *dach-1* dauers (n = 78) are more resistant to 0.5 mM trichlorfon compared to N2 dauers (n = 76). Error bar indicates SE. Statistical analysis is performed by Log-rank (Mantel-Cox) test.

Figure S5. *dach-1* is not involved in resistance to 7% ethanol. The ethanol-induced paralysis of *dach-1* dauers (red line, n = 95) is similar to that of N2 dauers (black line, n = 95).

Figure S6. Cholesterol depletion does not influence 0.1 mM aldicarb hypersensitivity at dauer. Aldicarb sensitivity was measured as the ratio of moving worms at 80 min. n = 3 for each experiment.

Figure S7. Nictation behavior and dauer formation in *dach-1*. (A) *dach-1* is not defective in nictation, a dauer-specific dispersal behavior. The nictation ratio of *daf-2;dach-1(ys51)* (n = 45) is comparable to that of *daf-2* mutant (n = 45), and the nictation ratio of *dach-1(ys51)* (n = 15) is comparable to that of N2 (n = 15). (B) *dach-1(ys51)* (n = 3) showed normal dauer formation compared to wild type (n = 3). Error bar indicates SE.

Figure S8. *dach-1* is not critical for stress resistance in a non-dauer stage, L3. (A and B) The heat (A) and osmotic stress (B) resistance of L3 stage *dach-1(ys51)* animals is comparable to that of L3 stage N2 (n = 3).
