# Supplementary figures and images for: A genetic screen for aldicarb resistance of Caenorhabditiselegans dauer larvae uncovers 2 alleles of dach-1, a cytochrome P450 gene
Source: G3 (Bethesda). 2022 Oct 4;12(12):jkac266. doi: 10.1093/g3journal/jkac266 (PMC9713407; doi:10.1093/g3journal/jkac266)

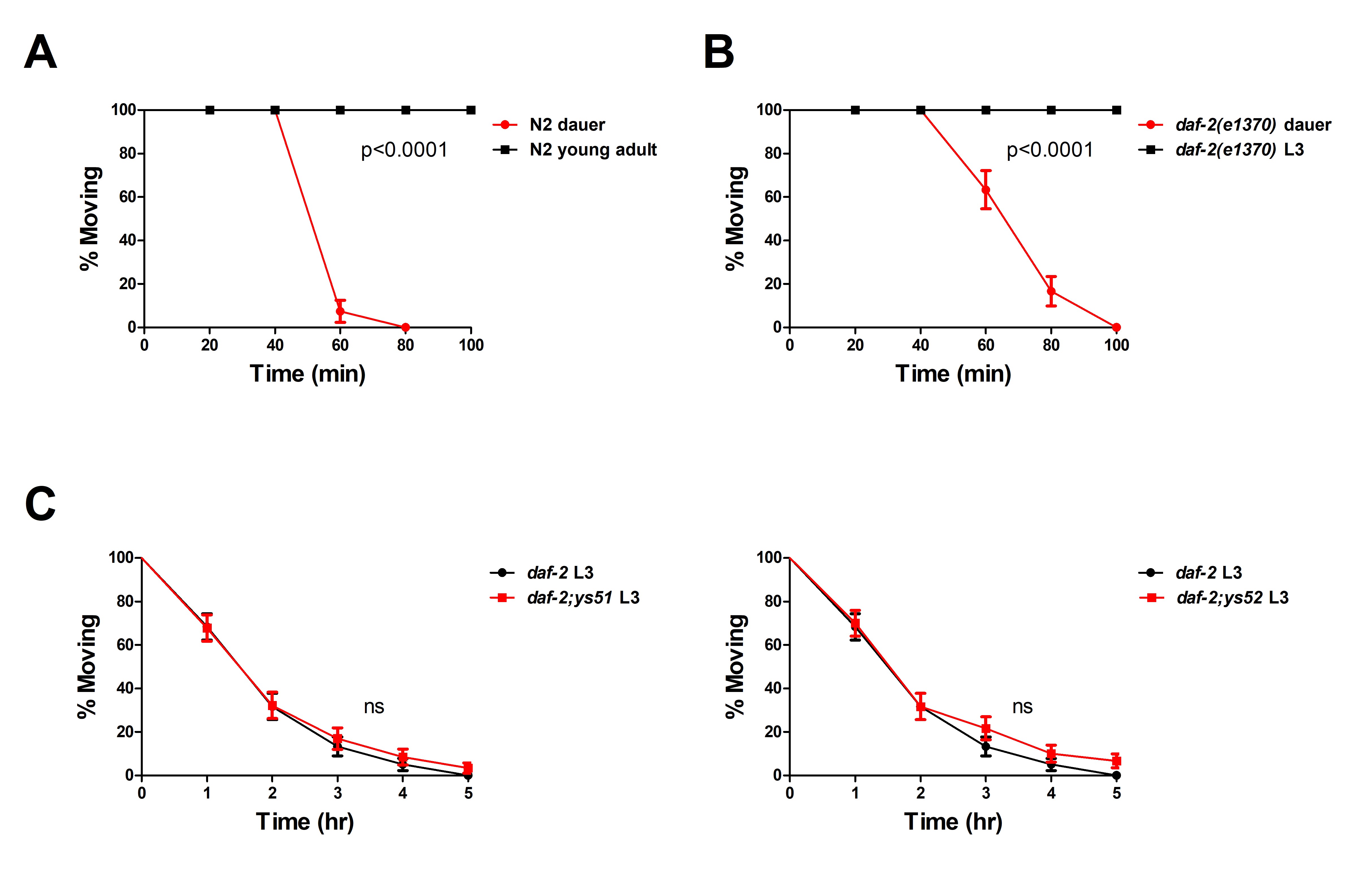

Supplement: jkac266_Supplementary_Figure_S1 [file jkac266_supplementary_figure_s1.jpeg]

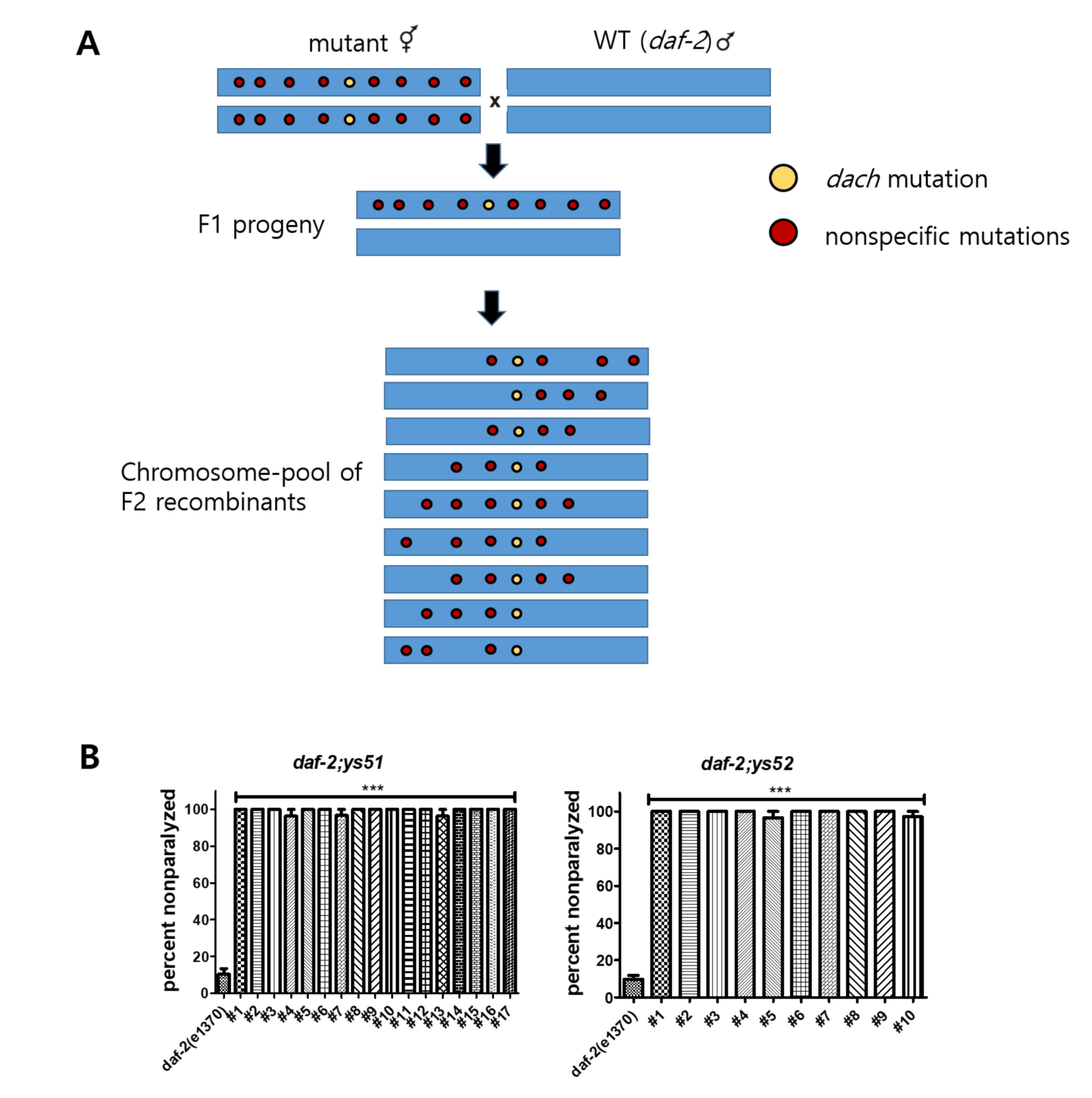

Supplement: jkac266_Supplementary_Figure_S2 [file jkac266_supplementary_figure_s2.jpeg]

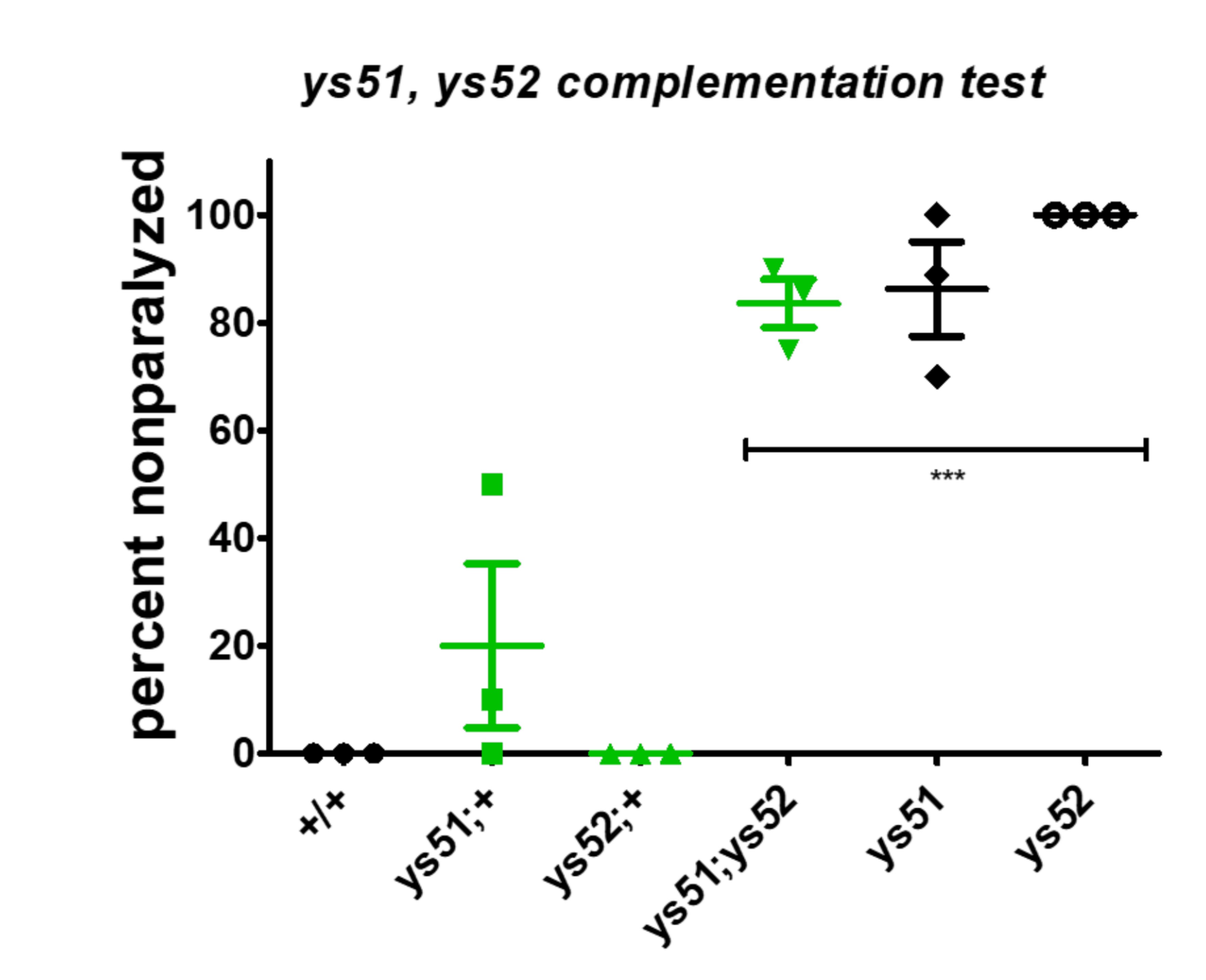

Supplement: jkac266_Supplementary_Figure_S3 [file jkac266_supplementary_figure_s3.jpeg]

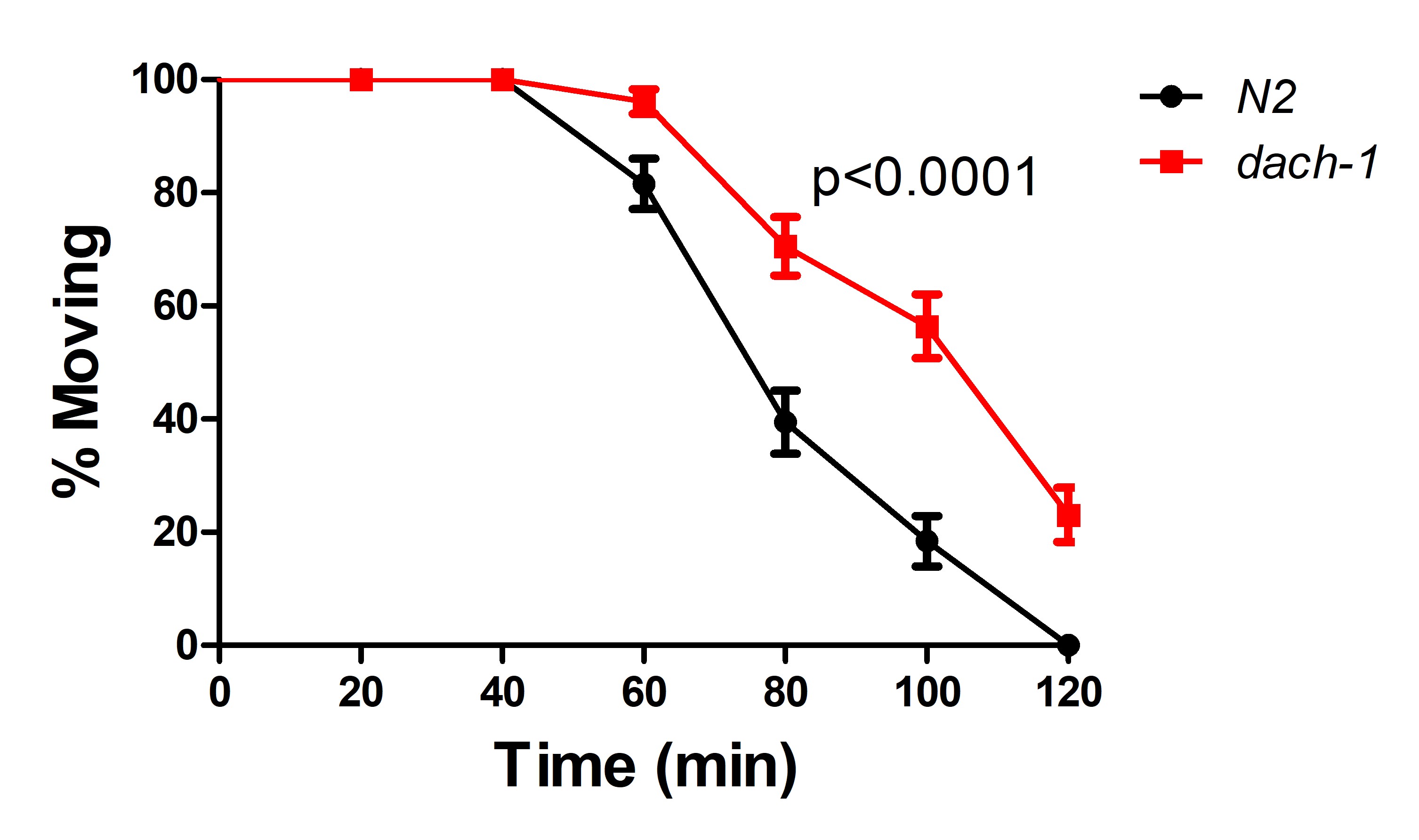

Supplement: jkac266_Supplementary_Figure_S4 [file jkac266_supplementary_figure_s4.jpeg]

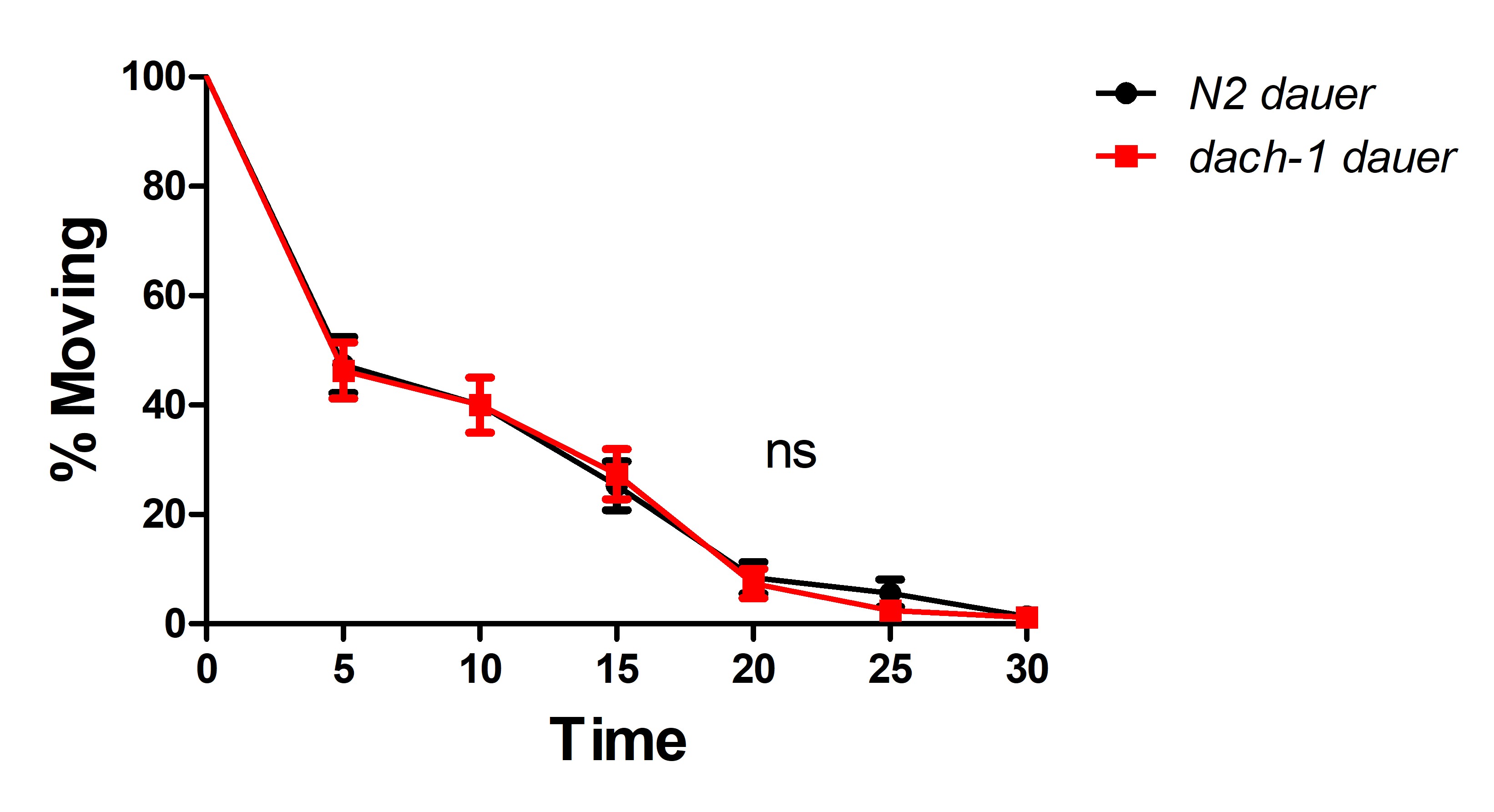

Supplement: jkac266_Supplementary_Figure_S5 [file jkac266_supplementary_figure_s5.jpeg]

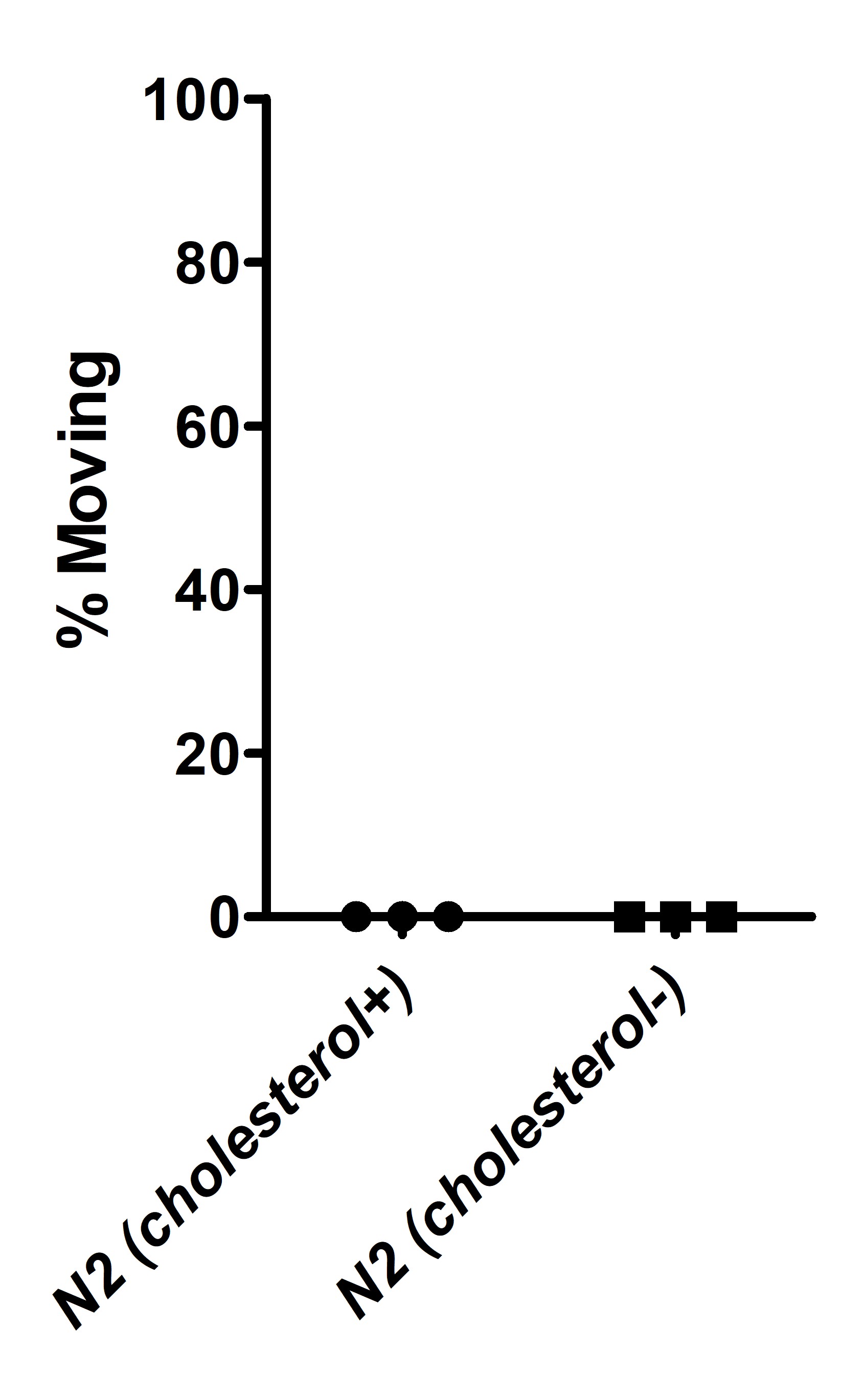

Supplement: jkac266_Supplementary_Figure_S6 [file jkac266_supplementary_figure_s6.jpeg]

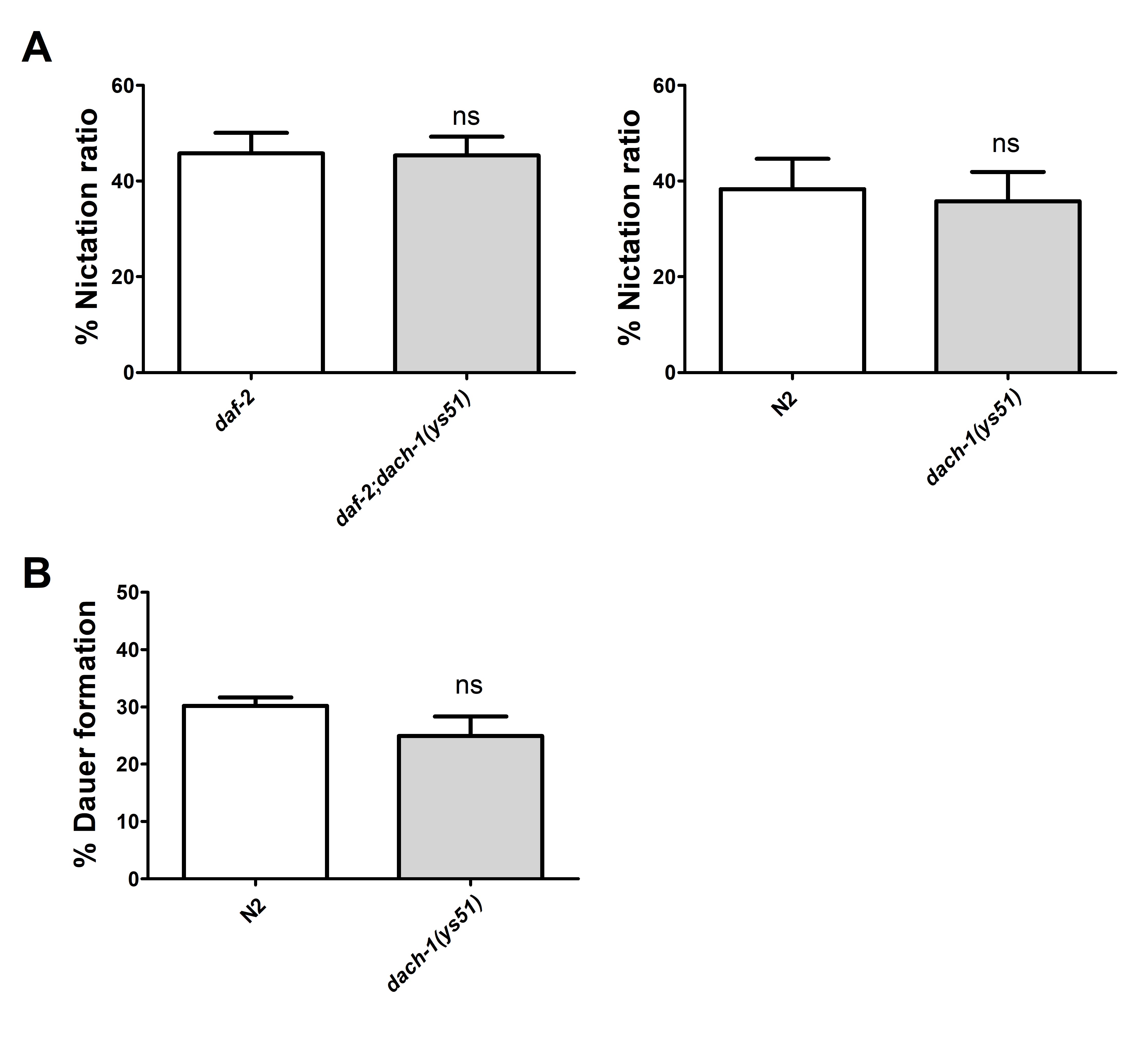

Supplement: jkac266_Supplementary_Figure_S7 [file jkac266_supplementary_figure_s7.jpeg]

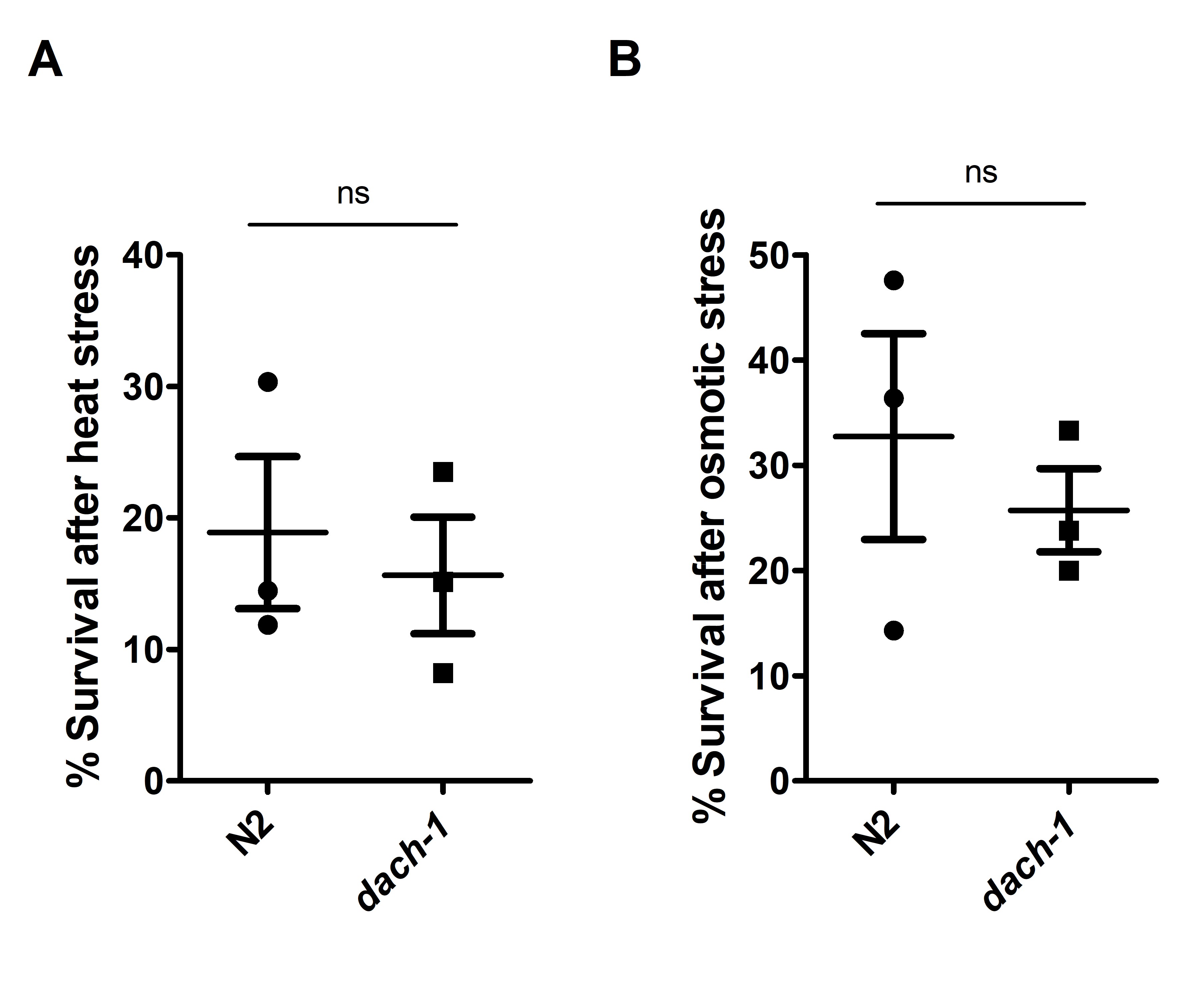

Supplement: jkac266_Supplementary_Figure_S8 [file jkac266_supplementary_figure_s8.jpeg]
